# The Role of Temporally Coarse Form Processing during Binocular Rivalry

**DOI:** 10.1371/journal.pone.0001429

**Published:** 2008-01-16

**Authors:** Jeroen J. A. van Boxtel, David Alais, Casper J. Erkelens, Raymond van Ee

**Affiliations:** 1 Department of Physics, Helmholtz Institute, Utrecht University, Utrecht, The Netherlands; 2 School of Psychology, University of Sydney, Sydney, New South Wales, Australia; University of Southern California, United States of America

## Abstract

Presenting the eyes with spatially mismatched images causes a phenomenon known as binocular rivalry—a fluctuation of awareness whereby each eye's image alternately determines perception. Binocular rivalry is used to study interocular conflict resolution and the formation of conscious awareness from retinal images. Although the spatial determinants of rivalry have been well-characterized, the temporal determinants are still largely unstudied. We confirm a previous observation that conflicting images do not need to be presented continuously or simultaneously to elicit binocular rivalry. This process has a temporal limit of about 350 ms, which is an order of magnitude larger than the visual system's temporal resolution. We characterize this temporal limit of binocular rivalry by showing that it is independent of low-level information such as interocular timing differences, contrast-reversals, stimulus energy, and eye-of-origin information. This suggests the temporal factors maintaining rivalry relate more to higher-level form information, than to low-level visual information. Systematically comparing the role of form and motion—the processing of which may be assigned to ventral and dorsal visual pathways, respectively—reveals that this temporal limit is determined by form conflict rather than motion conflict. Together, our findings demonstrate that binocular conflict resolution depends on temporally coarse form-based processing, possibly originating in the ventral visual pathway.

## Introduction

We addressed a central problem in perception: how to parse a continuous stream of information into meaningful events, resolve ambiguities, and shape awareness. Although the spatial determinants underlying these processes have been extensively studied using binocular rivalry [1]–[3], the temporal determinants have been largely unexplored. However, the time domain is important because under natural viewing conditions we make several saccadic eye movements each second. For this reason, incompatible binocular input may need to be resolved at the same rate.

The importance of the time domain is well established in binocular rivalry studies employing brief presentations of interocular conflict [4]–[9]. These studies showed that temporal offsets between two competing patterns strongly bias perceptual outcome. On the other hand, recurring temporal modulations during long-lasting interocular conflict have a rather limited effect. For example, interocular temporal frequency differences between spatially matched stimuli do not induce rivalry [10]. With the presence of interocular spatial conflict, unbiased rivalry may be instigated between stimuli that are presented intermittently, even when they are temporally out of phase and so have no temporal overlap [11]. These findings suggest that binocular rivalry has a certain insensitivity to recurrent temporal modulations.

Binocular rivalry is, however, not completely insensitive to the temporal lay-out of stimulation. When stimuli are shown intermittently at temporal frequencies below 3Hz, rivalry ceases to occur and the individual presentations from each eye are perceived [11]. Interestingly, this limit to rivalry is an order of magnitude larger than temporal resolution of the visual system [12], [13], and therefore reflects a relatively coarse temporal process. The temporal limit is in the range where eye movements generally dominate the temporal components of visual stimulation. Possibly, the 3Hz temporal limit reflects a functional adaptation of the visual system, which makes the visual system particularly insensitive to image changes that, under normal conditions, are most likely the result of eye movements.

The aim of the current study is to investigate the characteristics of this temporally coarse process that maintains rivalry when the individual rivalrous patterns are presented at rates as low as 3 Hz. To this end, we used the procedure of intermittent stimulation to induce rivalry [11]. A great advantage of employing temporal trains of discrete stimuli was that we had the possibility to manipulate various low-level visual features (such as, temporal offsets, contrast polarity, eye-of-origin information, and stimulus energy), while holding constant visual features related to form (such as orientation). We also examined whether the temporal limit for binocular rivalry requires form conflict, by employing stimuli that conflict in motion but not form. Comparing the role of form and motion, we may be able to assign the source of the temporal limit of rivalry to processing in the ventral and dorsal visual pathways, respectively [14].

## Results

### Determining the temporal limit of binocular rivalry

**Figure 1:**
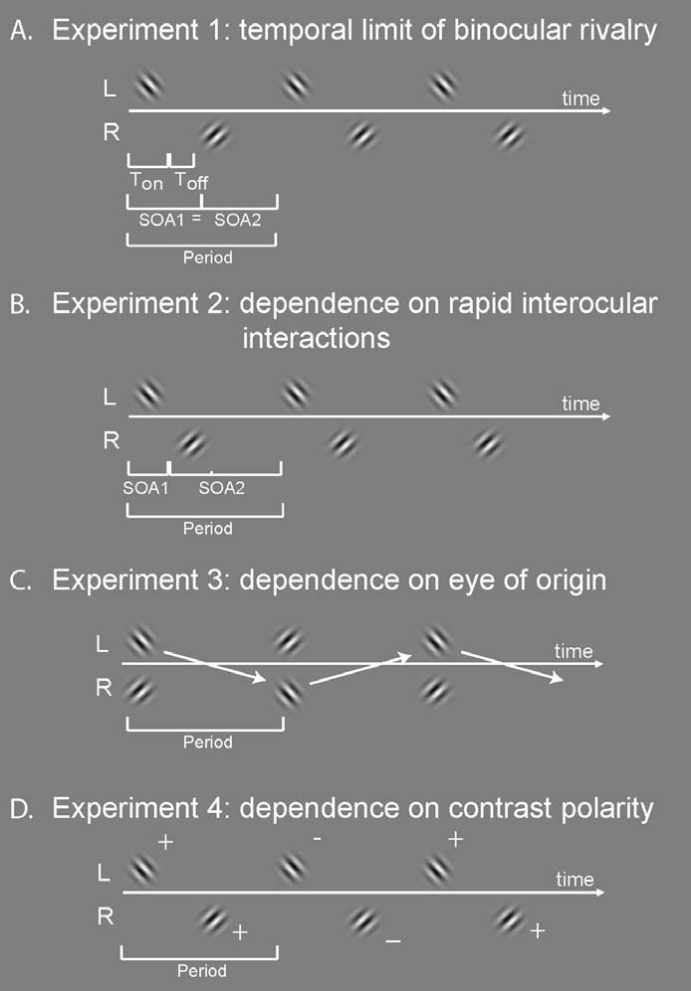
The temporal layout of the stimuli. A. In experiment 1, the temporal limit of binocular rivalry was determined. Stimuli were gabor patches of orthogonal orientation in the two eyes. T_on_ is the presentation duration of the patterns. T_off_ is the time between two presentations. T_on_ and T_off_ together make up the stimulus onset asynchrony (SOA), which is identical for the pattern sequence from left (L) to right (R) eye, and vice versa. B. Experiment 2: dependence on rapid inter-ocular interactions. Parameters as in experiment 1, except that the stimulus in R followed immediately the stimulus in L (or vice versa). Inserting various numbers of blank frames varied the period of repetition. C. Experiment 3: dependence on eye of stimulation. Stimuli in L and R are presented synchronously, but the orientations were swapped between the eyes on every new presentation (see arrows). D. Experiment 4: dependence on contrast polarity. Stimuli as in experiment 1, except that on every new presentation the contrast polarity was reversed (for illustrational purposes indicated with “+” and “−” signs).

We determined the temporal limit for inducing rivalry with temporally non-overlapping grating stimuli (Fig 1a). Brief grating presentations were delivered to the eyes in a repeating train of impulses, while varying the repetition period of the patterns between trials. Importantly, although the gratings were spatially conflicting, they were temporally interleaved between the eyes so that they were never simultaneously present [11]. With very long repetition periods, every flashed grating was perceived individually, so that the observer perceived rapid and regular switches in orientation. With very short repetition periods (i.e. high stimulation frequency, approximating continuous presentation), normal binocular rivalry was perceived, that is, a slow and irregular alternation between the two eyes' images. Between these qualitatively different perceptual manifestations, there was a transition-point indicating the temporal limit of binocular rivalry. We fit a curve through the data (see methods), and determined the transition point by finding the repetition period of the patterns at which the cumulative percept duration decreases to half the amount obtained with rapidly alternating stimuli. The average value of the temporal limit to rivalry was 377±55 ms (4 subjects, here as well as in the remainder of the article, these values refer to the mean±between-subject s.e.; R^2^-values of the individual fits: 0.99, 0.99, 0.92, 0.98), consistent with previous estimations [11] using similar stimuli. Next, we investigated what characteristics underlie the temporal limit to binocular rivalry.

### The independence from rapid inter-ocular interactions

**Figure 2:**
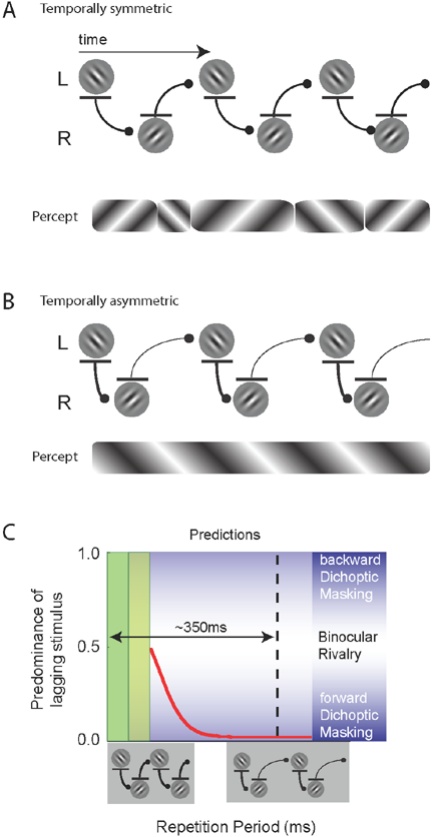
Descriptions of possible interactions between the two eyes' images and the predicted perceptual consequences, in experiment 2. A and B: interactions between temporally resolved patterns in the two eyes in cases of temporally symmetric (A) and asymmetric (B) stimulation. The presumed inhibitory interactions are drawn (lines with disk-heads), assuming inhibitory interactions are strongest at shorter and decrease at longer delays [7]. The strength of inhibition is indicated by the width of the lines. Below each panel are schematized representations of the predicted perceptual outcomes (time-scale is different from the upper images), showing that in (A), normal rivalry should be observed, whereas in (B) a strong bias is expected, based on the masking literacture (see text). C The predicted perceptual consequences of introducing asymmetric stimulation. The ratio of the percept duration of the lagging stimulus over total percept duration is shown on the y-axis (“predominance of lagging stimulus”). For temporally symmetric presentations (left, see icon bottom) the inhibitory interactions between the patterns are equal and therefore the percept should be unbiased (i.e. a bias of 0.5, as in Exp 1). For temporally asymmetric presentations (see icon on the bottom right), perception should be biased towards the temporally leading stimulus because its inhibitory forces are stronger than the inhibition it receives from the lagging stimulus (see B). The vertical dashed line represents the approximate position of the temporal limit found in Experiment 1. The dark and light green rectangles represent the presentation of the leading and lagging patterns, respectively.

One may assume that every flashed pattern in experiment 1 was parsed from the visual stream, especially given the temporal resolution of the visual system (psychophysically measured as high as 30–80 Hz [13]). Because the two inter-ocular stimulus onset asynchronies (SOA, see Fig 1) were identical, the inhibitory interactions between the two eyes were balanced, leading to binocular rivalry (see Fig 2A). Inter-ocular inhibition is, however, not always equally strong. The dichoptic masking literature shows that short interocular SOAs lead to strong suppression from visibility (i.e. inhibition) of the lagging pattern by the leading pattern [7], [8], [15], while longer SOAs lead to increasingly weak suppression [7], [8]. Therefore, if every flashed pattern was indeed parsed from the visual stream, the introduction of an asymmetry between the two SOAs should result in strong perceptual biases (Figure 2B). In order to test this prediction, we fixed one of the two SOAs to 50 ms, which should allow for strong inhibition of the leading pattern on the lagging pattern [7], [8], [15]. The other SOA was varied over trials. We predicted that rapidly alternating symmetric stimuli (see icon at the left-bottom of the Figure 2C) should result in a 0.5 predominance of the lagging stimulus, just as in Experiment 1. However, as soon as any asynchrony is introduced by increasing one of the two SOAs (icon on the right) a bias to the leading stimulus should be obtained.

**Figure 3:**
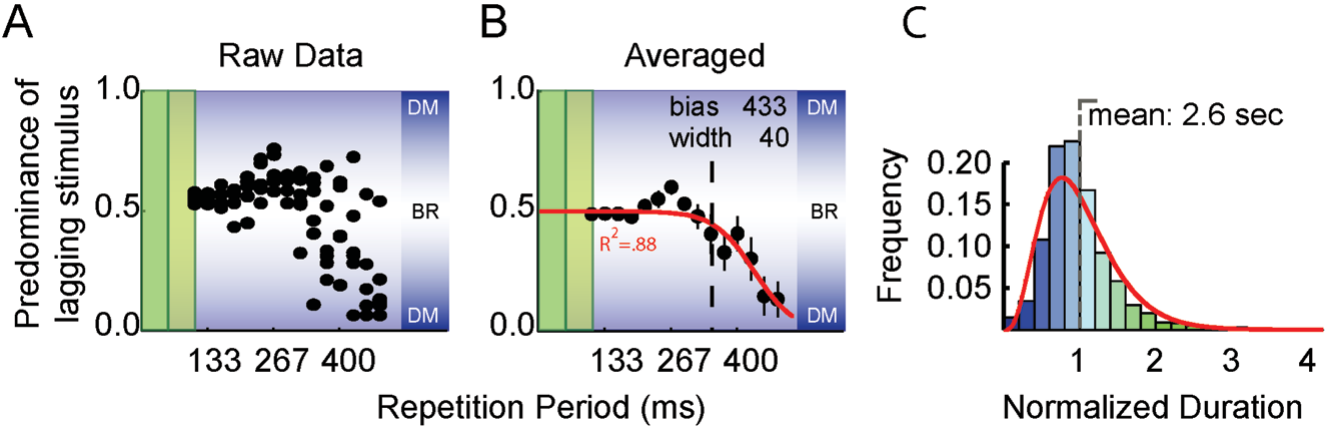
The independence of rapid interocular inhibition (Experiment 2). The temporal limit identified in Experiment 1 is largely uninfluenced by asymmetric stimulation between the eyes, and therefore independent of rapid inter-ocular inhibition. This subject's data show an unbiased perception for periods up to about 433 ms; only for greater periods inter-ocular inhibition causes perceptual biases. A. Data for single trials. B. Means (± s.e.m.). The vertical dashed line represents the position of the temporal limit found in Experiment 1. The small excursion towards unity near a period of 267 ms did not occur for other observers. Averaged over 4 subjects, the temporal limit is 348±33 ms (mean±between-subject s.e.). C. The dominance duration distribution for the conditions with repetition periods of 213–267 ms. Even though the patterns in the two eyes are not delivered simultaneously, a gamma distribution characteristic of binocular rivalry is obtained. Normalized dominance durations were obtained by dividing percept durations by the average percept duration for each subject individually. Gamma fit parameters: shape: 4.2; scale: 0.24. The non-normalized mean percept durations are also similar to durations for conventional binocular rivalry: 2.6±0.5 seconds (mean±s.e.m over subjects). BR = Binocular rivalry; DM = Dichoptic Masking (i.e. very biased rivalry), note that at long repetition periods the stimulus is indeed very much like a series of rapidly paced dichoptic masking trials.

**Figure 4:**
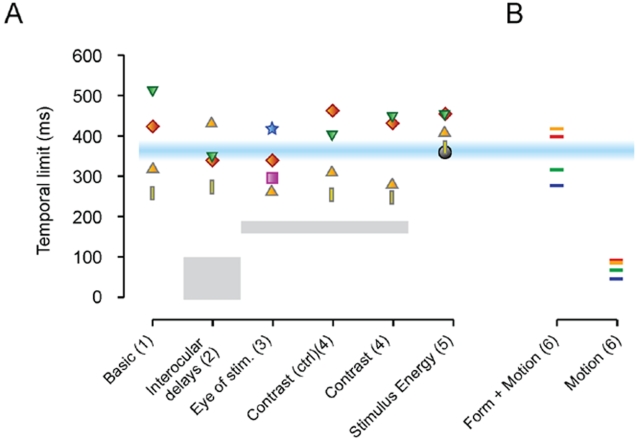
Overview of the obtained temporal limits. (A) Experiments 1-5. Individual subject data are denoted by different symbols. The label “Basic” refers to Experiment 1. Data in the Contrast (ctrl) condition was obtained with stimuli identical to those of experiment 1, which were interspersed with the stimuli of the contrast polarity experiment (“Contrast”, experiment 4). The numbers refer to the Experimental condition. The expected positions of the data points, if the experimental manipulations were effective, are indicated with grey boxes. The results show that the data cluster around a much higher temporal limit. The horizontal blue line represents the position of the estimated temporal limit, based on these experiments (363±11 ms, mean±s.e. over experiments 1-5). (B) Experiment 6. The Form+Motion conditions exhibited the same ∼350 ms temporal limits as Experment 1-5, while the Motion-only conditions did not.

Interestingly, and opposite to expectation, we observed that binocular rivalry was unbiased. This result was independent of the inter-ocular SOAs, and held at all repetition periods up to 348±33 ms (Fig. 3A,B)—very close to the limit found in Experiment 1. R^2^-values for the fits of the individual subjects are: 0.88, 0.98, 0.82, 0.87. Individual subject data on the temporal limit may be found in Figure 4. Note that this limit is significantly above the 50 ms limit that should be expected if rapid interocular interactions drove the inhibitory forces (p<0.002; one-tailed t-test). The perceptual dynamics gave average dominance durations typical of binocular rivalry with continuously presented stimuli (mean±s.e.m over subjects: 2.6±0.5 sec), as well as the characteristic gamma distribution of dominance times (see Figure 3C). Only at repetition periods that surpassed the temporal limit identified in Experiment 1, a definite bias towards the leading pattern was found.

### The independence from eye-of-origin information

In experiment 3, we investigated whether the temporal limit of binocular rivalry depends on the eye of origin of the competing patterns. Both eyes' patterns were briefly presented simultaneously (∼50 ms) and swapped between the eyes on every presentation [16], while varying the repetition period between trials (see Fig 1C). Research on related stimuli [16] suggests that rivalry may occur with our stimuli in combination with the swapping procedure. The temporal limits of this process have however not been compared to more conventional binocular rivalry stimuli. If the temporal limit with the swapping paradigm were to remain unchanged at around 350 ms, it would be dependent on the repetition frequencies of the patterns *irrespective* of the eye of origin. Alternatively, if the limit halves (∼189 ms, i.e. repetition needs to be twice as rapid as in Experiment 1), the temporal limit is dependent on the eye-specific repetition period of a pattern. The experiment showed that temporal limits were close to the values found in our other experiments (330±34 ms; R^2^-values of fits: 0.77, 0.83, 0.94, 0.98), and significantly above a limit of 189 ms (half the limit in exp 1; p<0.02), indicating that the temporal limit depends on pattern repetition frequencies irrespective of the eye of origin and irrespective of intra-eye repetition frequencies. Individual subject data on the temporal limit may be found in Figure 4.

### The independence from contrast polarity

In experiment 4, we used the paradigm of experiment 1, but now with gratings that were reversed in contrast polarity on every presentation (Fig 1D), thereby changing the luminance profile of the stimulus, but leaving pattern (i.e. orientation) information unchanged. If binocular rivalry were to depend on the repetition of *identical* stimuli within a *single* eye, repetition periods in this experiment should be halved. However, we again found a temporal limit not different from experiment 1 (352±49 ms [R^2^-values: 0.72, 0.97, 0.72, 0.97]; and significantly above 189 ms: p<0.025), again demonstrating a pattern-dependence. (Again, individual subject data may be found in Figure 4.)

### The independence from stimulus energy

**Figure 5:**
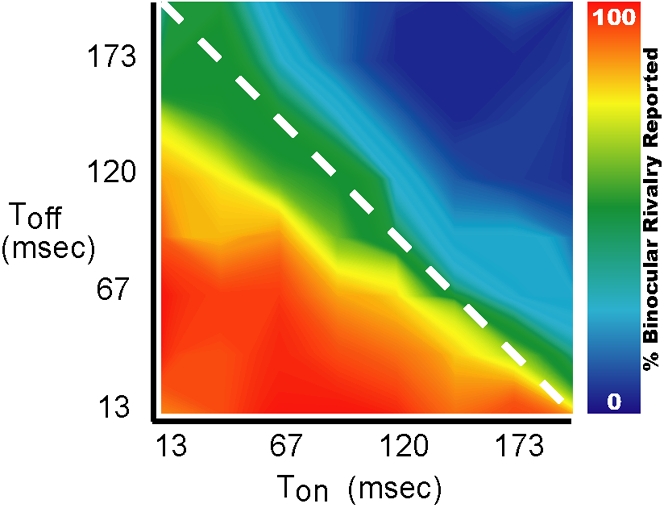
Independence from stimulus energy (Experiment 5). The results depict the percentage of trials per condition categorized as containing binocular rivalry, averaged over subjects. The ‘on’-times (T_on_) and ‘off’-times (T_off_) were independently varied between 13 and 200 ms, in steps of 26.6 ms. There was no significant dependence of the temporal limit ( = 2(T_on_+T_off_)) on the size of T_on_, and therefore on luminance (one-way ANOVA F(7,46) = 0.51; p>0.8). Instead the transition occurred at a repetition period of ∼400 ms (represented by the dashed line), irrespective of the size of T_on_.

In experiment 5, we investigated whether stimulus energy (i.e. luminance) would influence the size of the temporal limit. Luminance was varied by varying ‘on’ and ‘blank’ times from 13ms to 200 ms. The observers categorized 6-sec trials as containing rivalry or rapid and regular stimulus alternations. We observed that the limit was not dependent on the luminance energy of the stimuli, or equivalently on ‘on’ and ‘blank’ times (one-way ANOVA, F(7,46) = 0.51; p>0.8; Figure 5, individual subject data may be found in Figure 4), and thus the temporal parsing limit is not likely to be due to simple neural persistence or temporal integration. The mean temporal limit was: 410±19, R^2^-values of fits: 0.99, 0.999, 0.999, 0.74, 0.97, 0.997.

### A comparison of temporal limits

The previous experiments suggested that there is a single ∼350 ms pattern-based limit to rivalry. This suggestion was supported by a one-way ANOVA that revealed that the measured temporal limits across all experiments were not significantly different (F(5,24) = 0.52; p>0.75), and a post-hoc Tukey test showed no significant differences between individual experiments (Figure 4A). Note, the due to the variance between the subjects, we cannot exclude the possibility that, if more subjects were measured, we could find significant differences among the different experiments. However, the mean temporal limits of the different experiments are rather similar, and an effect size larger than about 50 ms is not expected (which will be small compared to the 350 ms temporal limit). This finding, together with the ANOVA results, and the finding that all experiments differed significantly from a limit of 189 ms (i.e. half the limit in experiment 1, all p<0.025, one-tailed t-test), suggest that there is by-and-large a single process responsible for most of the ∼350 ms temporal limit, which is a pattern-based process.

Combining the data over all the experiments, to obtain a better estimate of the temporal limit, we estimate the size of the temporal limit to be 363±11 ms (mean±s.e.m. over estimations from experiments 1-5).

### Dependence on form conflict; independence from motion conflict

These experiments have shown the importance of form information in binocular rivalry relative to low-level information. We investigated whether the temporal limit depends on form or motion conflict, as well.

**Figure 6:**
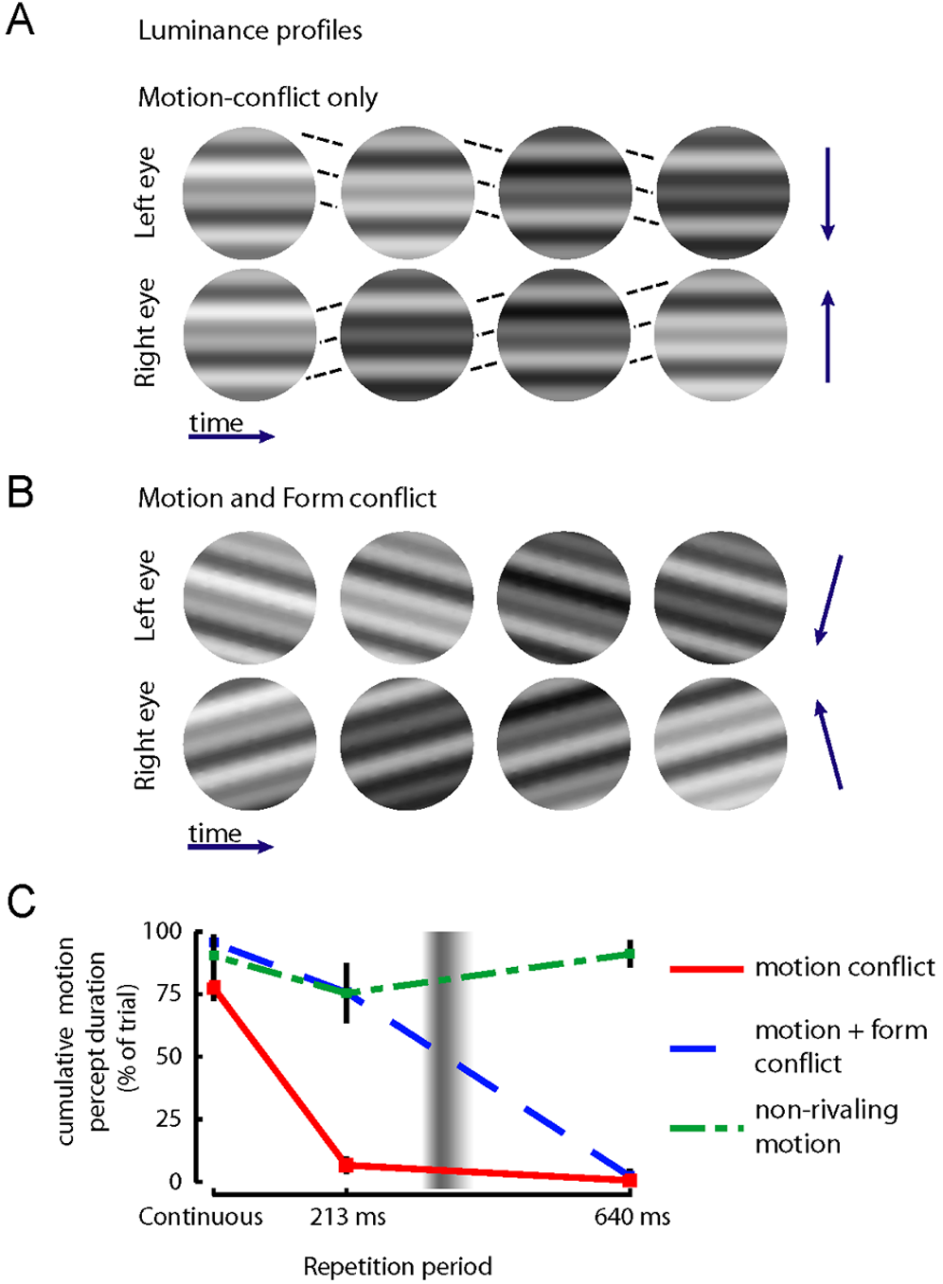
The temporal limit for binocular rivalry is preserved with form conflict, but not with motion conflict (Experiment 6). A. Stimuli for the motion-conflict-only conditions have identical orientation, but different motion directions in the two eyes (see arrows on the right and dotted lines that trace some of the edges' motions over time). B. Stimuli for the motion-and-form-conflict conditions have both different orientation and motion-direction. C. Rivalry ensues in the form-and-motion conflict condition for repetition periods smaller than ∼350 ms (blue dashed line), causing most of the trial being spent in one of two motion percepts. In the motion-only-conflict condition (red continuous line), rivalry was only observed for continuous presentations, and therefore it does not show the 350 ms temporal limit. At repetition periods of 213 and 640 ms, a static pattern was observed. Motion was, however, clearly perceived for all repetition periods when presented in non-rivalrous conditions (green dot-dashed line). Error bars are between-subject standard errors. The vertical Gaussian profile represents the estimated size of the temporal limit (363±11 ms; mean±s.e.m over experiments 1-5).

We constructed a novel binocular rivalry stimulus that allowed for the presence of motion conflict without form conflict (i.e. orientations were always matched). Stimuli consisted of three superimposed gratings of different spatial frequency (Fig. 6). Motion conflict was introduced by presenting both eyes with opposite motion directions (see the motion-conflict-only condition in figure 6A, and Movie S1). Form conflict was introduced by creating a 30-degree orientation conflict between patterns (see the motion-and-form conflict condition in figure 6B, and Movie S2).

When both orientation and motion direction were in conflict (blue line, Fig. 6C), we recovered the same ∼350 ms temporal limit that was revealed in Experiment 1-5 (despite the local luminance differences between the conflicting stimuli). Linear interpolation led to an estimate of 353±33 ms (mean±s.e. over subjects). However, when only motion information conflicted, rivalry was restricted to continuous presentations (red line, Fig 6C; linear interpolation led to a limit of 76±10 ms). Therefore, with motion-only conflict the temporal limit was not maintained. For the 213 ms and 640 ms repetition periods of motion-only conflict, subjects perceived a stationary grating, indicating that motion information from both eyes was integrated. The absence of motion perception in these cases was not the result of the flicker in the stimuli or from impoverished motion signals, as unambiguous motion (i.e. the same motion in both eyes) led to clear motion percepts (green line, figure 6C). Individual subject data are shown in figure 4B.

## Discussion

It has been well-studied how binocular rivalry depends on spatial parameters [2], [3], [17]. Although generally studied with continuously present stimuli, rivalry also occurs when the two eyes' images are not delivered simultaneously [11], or when they are flickered at rate as low as 3 Hz (i.e. repetition periods of ∼350 ms) [11], [18]. Instead of being an oddity of the visual system, this phenomenon may reveal a key constituent of the visual processing, e.g. the temporal constraints on binocular conflict resolution.

A phenomenological description of the percept associated with our stimuli reveals two distinct time scales of temporal resolution in the visual system. On the shorter time scale, the brief stimulus presentations of ∼50 ms or so are perceived clearly as distinct stimulus pulses, as would normally be expected, given the visual system's temporal resolution. However, another longer time scale of approximately 350 ms is revealed when these stimuli are in interocular conflict. In this case, we observe binocular rivalry between flickering patterns, with perceptual alternations between the two eyes' images at a rate much slower than the flicker rate of the stimuli [11]. Only, when the 350 ms temporal limit for rivalry was surpassed, each individual stimulus in the alternating monocular presentation was perceived as an independent perceptual event.

We have investigated what upheld the rivalry in the absence of direct spatial and temporal conflict. We started the investigation with several low-level properties of the binocular conflict, as one of the current theories suggests that rivalry is a process of low-level conflict resolution [19].

First, we introduced differential interocular delays between the presentations of the conflicting patterns. This manipulation should produce strong perceptual biases towards the pattern that is followed by the shortest interocular delay, because interocular inhibition is strong at short delays and weakens at longer delays [7], [8], [15]. However, we found that over a large range of stimulus repetition periods, binocular rivalry occurred with normal perceptual dynamics (Fig 3). The lack of bias suggests that the temporal limit for rivalry is not dictated by rapid inter-ocular inhibition, consequently models of binocular rivalry that depend on rapid interocular interactions (e.g. [15]) cannot capture our results. Strongly biased rivalry, i.e. dichoptic masking [20], was instantiated only when the temporal limit of binocular rivalry (at ∼350 ms) was passed (Fig 3). Incidentally, this finding also shows that temporal modulations do not necessarily prevent rapid interocular interactions [cf. 15].

The temporal limit of rivalry remained unchanged as well when the two eyes' patterns were swapped between the eyes after every presentation (experiment 3; Fig 4). This finding shows that the temporal limit to rivalry is largely indifferent to the eye of origin of the stimuli, i.e. the limit is upheld by a binocular and pattern-based process. Consistent with this conclusion is our recent finding [21] that monocular rivalry, which is necessarily pattern-based, shows this same temporal limit.

Luminance integration, as a low-level visual process, might be responsible for our results. However, we showed that contrast polarity reversals, which should have weakened stimulus strength and therefore rivalry if luminance integration occurred, had no effect on the temporal limit (experiment 4; Fig 4). Nor did higher stimulus energies, or shorter ‘blank’ periods, lead to longer temporal limits (experiment 5; Fig 4, 5). Luminance integration may therefore be excluded as a possible mechanism underlying the temporal limit to rivalry.

These data suggest that an essential feature for the maintenance of rivalry with intermittent stimulation is the repetition of *pattern* information within a temporal window of ∼350 ms. Therefore, the temporal limit might be a reflection of the temporal grain of the visual system's ventral pathway, which is important in form processing [14]. Indeed, we found that the ∼350 ms limit was maintained with form-based conflict, but not with only motion conflict (Figure 4B & 6). Since form information is mainly processed in the ventral visual pathway, and motion information—when not defining a form—is mainly processed in the dorsal pathway [14], these findings show that the temporal limit is not a feature of the motion pathway, and may indeed be a characteristic of the ventral form pathway. This finding is consistent with the report [22] that rivalry more strongly affects the ventral than the dorsal visual pathway.

Overall, the data suggest that the ∼350 ms limit to binocular rivalry is a result of a temporally coarse form processing at a binocular level. What is essential to the maintenance of rivalry in the temporal domain is the *repetition* of each of the rivalrous patterns within a 350 ms time window from their last presentation. The inhibitory interactions that cause the percept to alternate between the two eyes' views operate on these temporally coarse form representations (as clearly suggested by Experiment 2 and 3). The resulting rivalry is characterized by the same dynamics as conventional binocular rivalry (Figure 3).

There is an interesting link to be made here to visible persistence [23] (or ‘gestalt fusion’ [13]). Visible persistence shares a key characteristic with the temporal limit to binocular rivalry: storage of visual information over long blanks periods. A pattern that is repetitively presented and blanked may be perceived to be continuously presented when blanks are smaller than ∼250–500 ms [e.g.], [13], [23], [24,25]. This effect resembles our finding of continued rivalry in the temporary absence of visual stimulation. Consistent with our data, visible persistence is thought to depend on cortical processes [24], [26]–[28].

As an aside, our results also bear on the still unresolved debate of whether pure motion conflict may result in rivalry [29]–[32]. We found that motion rivalry was rare and depended on continuous stimulus presentations, while form rivalry was induced whenever stimulus repetitions followed each other within 350 ms. These data show that motion-based rivalry has temporal characteristics different from form-based rivalry. Our results, furthermore, suggest that motion processing during rivalry is subservient to form processing. When form rivalry occurred, only the motion of the dominant orientation was perceived. When form-based rivalry was absent, motion information from both eyes was integrated. These results suggest that form information may determine whether interocular integration or segmentation of motion signals occurs (but see exceptions in [33]–[35]). These results therefore extend previous studies that showed the governing power of form on motion processing [36]–[38].

At what level within the visual system is the temporal limit to rivalry determined? In the binocular rivalry literature, which is based on studies using continuously present stimuli, there has been a lively debate about what mechanisms maintain rivalry, and at what level rivalry takes place. Some have argued that rivalry takes place early in the visual cortex (e.g. [19], [39], [40]), and that it depends primarily on low-level stimulus characteristics. Meanwhile, others have maintained that rivalry takes place in higher visual areas [16], [41], [42], involving stimulus-based rivalry. Recently, it has been proposed that several levels within the visual system may contribute to the process of rivalry [15], [17], [43].

To some extent, our data are consistent with studies that implicate early visual areas in the process of rivalry [39], [40]. For instance, the temporal limit of binocular rivalry has several characteristics that are consistent with activity patterns of (binocular) complex cells in the primary visual cortex, e.g. independencies of eye-of-origin information, and contrast-polarity, and a pattern-selectiveness of adaptation [44]–[47].

Nevertheless, the temporal constraint on binocular rivalry (the 350 ms form-repetition limit) has a rather coarse temporal resolution. Since the 350-ms limit is an order of magnitude greater than the critical flicker fusion frequency [13], the limit seems rather long for early visual areas. Some have argued that rivalry occurs mainly in the parvocellular (P) system [30], because the P system shows a sustained activity compared to the magnocellular (M) system's transient response [30]. However, the P system is not nearly as sustained as would be needed to bridge blank periods exceeding 300 ms [26], [48], [49]. Therefore, the P system, at least at early visual levels, does not seem to be fully responsible for the 350 ms temporal limit. Consequently, our data suggests that at least part of the network involved in determining the *temporal* constraints on rivalry is situated at levels higher than V1 (complex) cells. This conclusion may, or may not, extend to binocular rivalry with continuously presented stimuli. If it does, the frequently reported eye-based processes during binocular rivalry (e.g. [50]–[53]), may be (partly) explained by means of feedback [17], [54].

In any case, we show that the temporal limits to binocular rivalry are determined at a binocular pattern-based level. A possible candidate area for the identified temporal limit is the lateral-occipital cortex (LOC) in the ventral form pathway, which is important in shape and object processing [14]. The human LOC, but not lower visual areas, shows prolonged activity up until about 400ms after brief stimulation [26], [55], and it is modulated according to the percept during binocular rivalry [56]. Moreover, the possibly related phenomenon of visible persistence, which has similar temporal constraints [13], [24] is localized in the LOC [26]–[28]. A possible objection to our suggestion is that binocular rivalry is strongly depended on spatial alignment of the stimuli, which is at odds with relatively large receptive field sizes of LOC neurons (in humans, estimated 5× the size of V1/V2 neurons [57]). However, the temporal constraints on rivalry, reported on in this report, may be determined in other cortical areas than the spatial constraints (cf. [58]), consistent with the idea that rivalry is a multi-leveled, and multifaceted process [17]. Additionally, the combined activity of several LOC neurons may produce spatial sensitivity much higher than the individual neurons themselves (cf. [59]).

What might be the use of the temporally coarse form processing during rivalry? We suggest it may be related to the frequency of saccadic eye movements, which occur around 3 times per second. In the interests of temporally stable perception, a perceptual interpretation, once formed, should be stable over several fixations. We have found that at the time scale of about 350 ms, binocular conflict resolution is greatly influenced by temporal long-range interactions of pattern information, which provided a stable perceptual interpretation spanning several stimulus presentation cycles. Such form-based interactions may ensure that we perceive a stable world in the face of fixation changes and eye-blinks. Our data suggested that this mechanism is part of the visual “what” or form pathway. This suggestion dovetails well with the finding that during saccades “where” information (from the dorsal pathway) is suppressed whereas “what” information (from the ventral pathway) is retained [60], preserving form information over saccades. Moreover, it seems that the 350 ms limit is not specific to binocular rivalry but extends to binocular fusion processes. It has been shown that binocular depth perception remains stable when the simuli carrying the depth information are presented intermittently with temporal gaps of up to ∼350 ms [61]–[63]. These data provide further support that the coarse temporal processing is useful for the perceptual stability in face of frequent saccadic eye-movements. Outside the laboratory setting, the functioning of this system would be aided if similar orientations are fixated before and after saccades, which seems to occur quite naturally, as saccades are generally small [64], [65] and neighboring patches in visual scenes have very similar orientation content [66].

### Conclusion

Although the spatial determinants of rivalry have been well characterized, the temporal determinants have been largely neglected. We are the first to systematically investigate what underlies the temporal limits to binocular rivalry. We have revealed a binocular, pattern-based, and temporally coarse mechanism, possibly positioned in the ventral form-pathway, that is an essential and heretofore unrecognized mechanism in the formation of visual awareness during the resolution of binocular visual conflict when processing dynamic, inherently-ambiguous, visual information.

## Methods

### Apparatus

Images were presented on a 22″ LaCie electron22blueIV monitor (1600×1200 pix, refreshed at 75 Hz). Subjects were seated at 46 cm from the screen, using a chin-rest to stabilize head position. Experimental procedures were reviewed and approved by the Institutional Review Board.

### Stimuli

Stimuli in experiments 1-5 were orthogonally oriented (45 deg from vertical) gratings, having a spatial frequency of 0.87 deg/cycle. The stimulus was seen through a circular Gaussian window (sigma = 0.76 deg, cut-off at a diameter of 4.5 deg). Michelson contrast was 1 at the stimulus center. Background luminance was 20.0 cd/m^2^, maximum and minimum luminance (i.e. white and black parts of the grating), were 71.9 cd/m^2^, and ∼0.0 cd/m^2^, respectively. The polarity inversion experiment was performed on a gamma-corrected monitor (background: 6.4 cd/m^2^; maximum luminance: 12.8 cd/m^2^; minimum luminance: ∼0.0 cd/m^2^). To aid in binocular fusion, the grating stimulus was surrounded by an annulus that could be seen by both eyes. It consisted of 20 equally 20 equally-sized parts alternatively made of full contrast (white) and zero contrast (grey). The annulus was 0.1 degrees wide, with a radius of 2.46 deg. Temporal characteristics of the stimuli are described in the main text and in Fig 1. Trials lasted for 60 seconds in experiments 1, 2, and 6; 30 seconds in experiments 3 and 4; and 6 seconds in experiment 5. Experiments 3, 4 and 6 had a small fixation mark. In experiments 1-4, individual events (T_on_) lasted for 4 frames (∼53 ms), followed by a variable blank period (T_off_). In experiment 5, T_on_ and T_off_ were both varied.

In Experiment 6, stimuli consisted of the sum of three gratings (0.11, 0.44 and 0.87 cycles/deg, the middle frequency was offset by half a cycle). The stimuli were seen through a Gaussian annular window that had a radius of 1.33 degrees and a sigma of 0.34 degrees, which left the surroundings of the fixation mark empty. Stimulus motion was introduced by phase-shifting each grating at every frame by 1/32 cycle. Stimuli were displayed for 8 frames (about 100 ms), before being blanked for 0 ms, ∼113, and ∼540 ms, after which the motion sequence resumed where it had left off. This cycle was repeated until the end of a trial, leading to, respectively, continuous presentations, repetition periods of about 213 ms, and 640 ms. Although luminance profiles of the eyes' patterns differed in the motion-conflict-only condition, this does not necessarily lead to rivalry [29], and luminance differences do not prevent patterns from being processed as identical forms when orientations are the same (as shown in experiment 4). The motion-and-form conflict condition was identical to the motion-conflict-only condition, except for a 30 degrees orientation conflict between patterns. The orientations were (near) horizontal to prevent binocular fusion and a resulting depth perception. Subjects indicated the direction of motion, ignoring form rivalry when it occurred without motion rivalry (which actually did not occur).

### Procedure

In experiments 1-4 and 6, subjects (n = 4) presses either of two keys to indicate their dominant percept, and were asked not to press when a fast and regular switching of the stimuli was perceived, or when the two patterns were overlaid without any of the two being stronger. In experiment 5, each trial was categorized as containing rivalry, or containing rapid and regular orientation switches. Subjects (n = 5) based their categorization on the last 3 seconds of the trial.

The psychophysical data were fitted with: *f*(*x*) = *a*/(1+*e* ^(*x*−*t*)/*w*^), where *a* was set to the maximum fraction of “cumulative percept duration/trial duration” per experiment (except in experiment 2, where *a* was 0.5), *w* is the width of the curve, and *t* is the temporal limit, which we report on in this study.

## Supporting Information

Movie S1 A schematic representation of the motion-only conflict stimuli with continuous presentations.
(0.05 MB MPG)

Movie S2 A schematic representation of the Motion and Form conflict stimuli with continuous presentations.
(0.06 MB MPG)
